# Male sex and iron deficiency risk at 6 months: the mediating role of rapid weight gain

**DOI:** 10.3389/fnut.2026.1829613

**Published:** 2026-04-23

**Authors:** Dongmei Ge, Jing Nie, Yanyan Shi, Chunrong Li, Wei Li, Jing Liu

**Affiliations:** 1 Department of Child Health Care, Sichuan Provincial Women's and Children's Hospital, The Affiliated Women's and Children's Hospital of Chengdu Medical College, Chengdu, Sichuan, China; 2 Department of Clinical Laboratory, Sichuan Provincial Women's and Children's Hospital, The Affiliated Women's and Children's Hospital of Chengdu Medical College, Chengdu, Sichuan, China; 3 Department of Obstetrics, Sichuan Provincial Women's and Children's Hospital, The Affiliated Women's and Children's Hospital of Chengdu Medical College, Chengdu, Sichuan, China; 4 Center of Women’s and Children’s Health Medical Research and Technology Innovation, Sichuan Provincial Women’s and Children’s Hospital, The Affiliated Women’s and Children’s Hospital of Chengdu Medical College, Chengdu, Sichuan, China

**Keywords:** infants, iron deficiency, mediation effect, sex, weight gain

## Abstract

**Background:**

Male infants exhibit a higher risk of iron deficiency (ID) than females, yet the underlying mechanisms remain unclear. Given that rapid postnatal growth may deplete iron stores and males typically experience faster early development, this study aimed to test whether rapid weight gain (WG) during the first 6 months mediates the association between male sex and ID risk at 6 months.

**Methods:**

We conducted a retrospective cohort study of healthy term infants born at Sichuan Provincial Women’s and Children’s Hospital in Chengdu, China, between January 2023 and June 2024. Associations among sex, WG, and ID were examined using logistic regression, adjusting for selected covariates and feeding type. Mediation analysis used SPSS PROCESS macro (Model 4) to estimate the indirect effect of sex on ID via WG with bootstrapped 95% confidence intervals.

**Results:**

This study included 355 infants (55.5% male), with a median WG of 4.9 kg. Total ID occurred in 22.5% of infants, with the highest incidence observed in exclusively breastfed males (36%). After adjusting for covariates and feeding type, male infants exhibited greater WG than females (*β* = 0.61 kg, 95%CI: 0.44 to 0.78, *p* < 0.001) and had higher odds of ID (aOR = 2.13, 95%CI: 1.23 to 3.70, *p* = 0.007). Further adjustment for WG attenuated the sex-ID association (aOR = 1.57, 95%CI: 0.88 to 2.83, *p* = 0.129), while WG remained independently associated with ID risk (aOR = 1.63 per kg, 95%CI: 1.17 to 2.27, *p* = 0.004). Mediation analyses revealed that WG partially mediated this association (indirect effect = 0.30; bootstrapped 95% CI: 0.11 to 0.54), accounting for 40% of the total effect.

**Conclusion:**

Male infants exhibited elevated ID risk at 6 months, with postnatal greater WG partially explaining this association. Our findings underscore the need for sex-specific guidelines on the introduction of complementary foods and on iron supplementation, particularly for rapidly growing, exclusively breastfed males before 6 months. Prospective trials should evaluate iron dosing strategies aligned with growth trajectories before clinical implementation.

## 1 Introduction

According to the World Health Organization (WHO) report, the global prevalence of anemia in children aged 6–59 months is 39.8%. Among them, approximately a quarter to half are associated with iron deficiency (ID) (1, 2). In China, national surveys show ID prevalence in children under 7 years decreased from 40.3% in 2000–2001 to 28.3% in 2022, while iron deficiency anemia (IDA) declined from 7.8 to 3.9% (3, 4). Despite this progress, ID remains prevalent in over one-quarter of children, with non-anemic ID emerging as the predominant concern (4). Even without anemia, chronic ID causes irreversible neurological impairment, compromising cognitive and behavioral development (5–9). While prematurity and low birth weight are well-established risk factors necessitating routine iron supplementation (10), ID in term, normal birth weight infants, who constitute the vast majority of births, represents a significant but underrecognized public health challenge with distinct etiological pathways.

Emerging evidence suggests that even among healthy term infants, specific subpopulations face disproportionate ID risk. Male sex has been increasingly recognized as an independent risk factor, with male infants demonstrating 1.5- to 3-fold higher ID rates compared to females (11–13). The biological mechanisms underlying this disparity remain incompletely understood but may relate to differential growth trajectories. Rapid early weight gain, frequently observed in male infants, represents a plausible mechanistic link between male sex and ID risk through hemodilution and expansion of blood volume that outpaces iron storage mobilization and dietary intake (14). While both male sex and weight gain independently predict ID (15), whether weight gain statistically mediates the sex-ID association remains unclear, especially at 6 months, a critical period when endogenous iron stores become depleted, and supplementation decisions are most debated. Compounding this risk, exclusive breastfeeding beyond 4 months, while optimal in many respects, provides insufficient bioavailable iron (0.2–0.5 mg/L) to meet the accelerated demands of rapidly growing infants (16), yet current guidelines reflect significant uncertainty regarding iron supplementation for term, normal birth weight infants. The American Academy of Pediatrics (AAP) recommends 1 mg/kg/day iron supplementation for exclusively breastfed infants starting at 4 months (17), whereas the European Society for Pediatric Gastroenterology, Hepatology, and Nutrition (ESPGHAN) does not endorse routine supplementation (18). Chinese guidelines similarly diverge, with some consensus statements recommending targeted supplementation for high-risk groups while others advocate universal approaches (10, 19–22). This lack of consensus highlights a critical research gap in the absence of risk-stratified, evidence-based recommendations to identify which breastfed infants truly require early iron intervention.

Term infants’ iron stores become depleted by 6 months, the age when China’s National Health Commission recommends routine hemoglobin screening (23), creating a critical window for detecting deficiency. We hypothesize that rapid weight gain (0–6 months) mediates the association between male sex and ID risk at this pivotal age in term, normal birth weight infants. Clarifying these pathways could identify high-risk infants, particularly males with accelerated growth, who would most benefit from targeted supplementation between 4 and 6 months. This study aims to: (1) quantify the association between male sex and ID risk at 6 months; (2) determine whether rapid weight gain mediates this association, with feeding practices adjusted as potential confounders. Our findings will inform precision public health strategies for iron supplementation in early infancy among Chinese infants.

## 2 Methods

### 2.1 Study design and population

**Figure 1:**
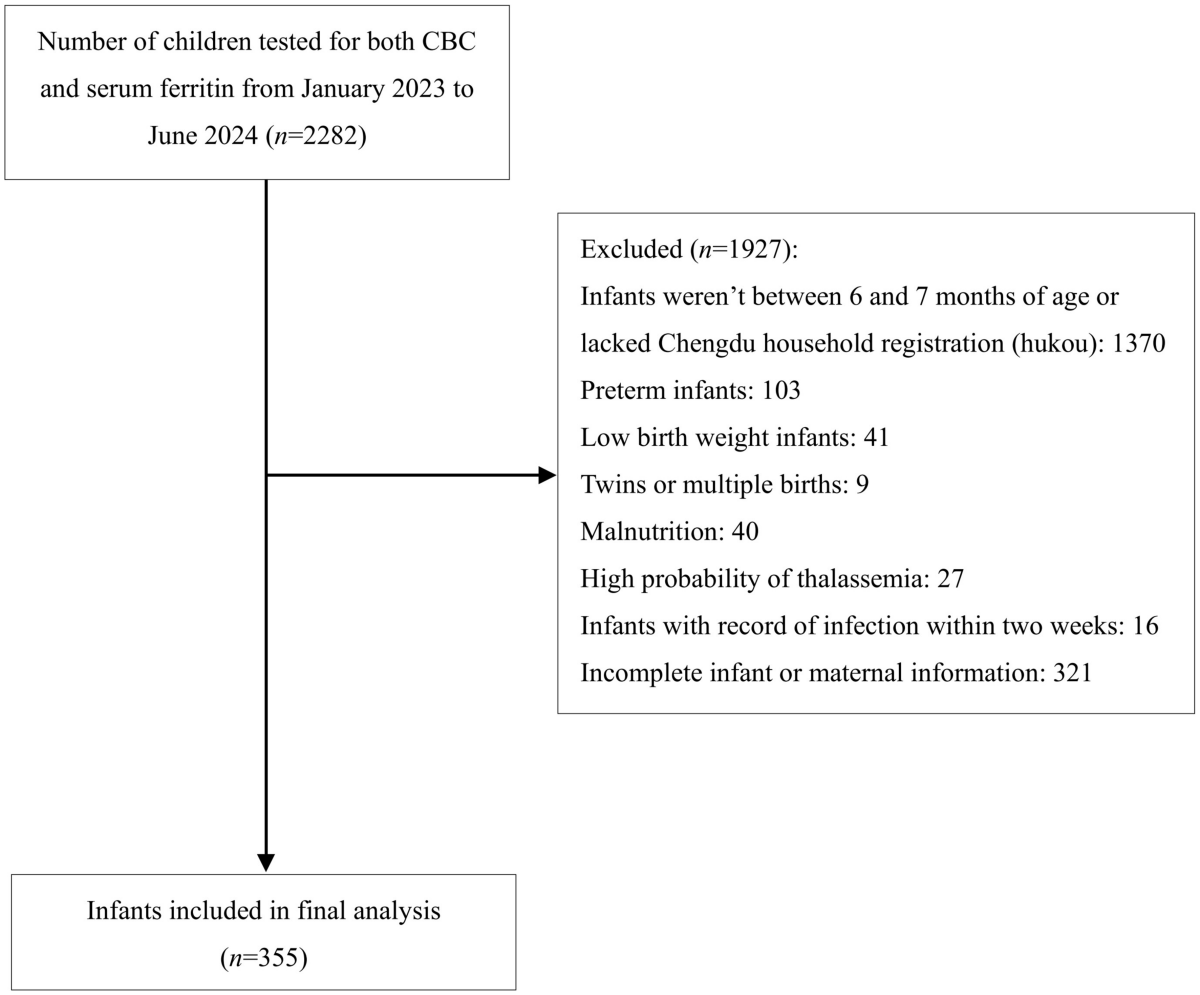
Flowchart of the participants selection.

We conducted a single-center, retrospective cohort study at Sichuan Provincial Women’s and Children’s Hospital, a tertiary care facility in Chengdu, China. Potentially eligible infants who had undergone both complete blood count (CBC) and serum ferritin testing between January 2023 and June 2024 were identified through electronic health record (EHR) review (Figure 1).

Inclusion criteria were as follows: (1) infants aged 6 to under 7 months; (2) healthy singleton infants born at term (37 ~ 42 weeks’ gestation and birth weight > 2,500 g); (3) no supplementation with iron preparations, human milk fortifiers, complementary foods, or nutritional supplements recorded in the outpatient medical record system; (4) absence of congenital disabilities, chronic diseases, or genetic metabolic disorders; (5) no recorded infections within the preceding two weeks, with white blood cell counts ranging from 5.0 to 14.2 × 10^9^/L, absolute neutrophil counts from 0.8 to 6.1 × 10^9^/L, and absolute lymphocyte counts from 2.8 to 10.0 × 10^9^/L (24); (6) with Chengdu household registration. Exclusion criteria included: (1) malnutrition (child malnutrition for aged 6 months encompasses stunting, wasting, and underweight conditions. Stunting is defined as a height-for-age Z-score (HAZ) less than −2 standard deviations (SDs), underweight as a weight-for-age Z-score (WAZ) below −2 SDs, and wasting as a weight-for-height Z-score (WHZ) less than −2 SDs) (25); (2) Infants with hemoglobin (Hb) < 110 g/L were evaluated using the Mentzer index (mean corpuscular volume/red blood cell count [MCV/RBC]) (26); those with index values <13, suggestive of high probability of thalassemia, were excluded; (3) presence of comorbid conditions potentially affecting iron metabolism, such as cardiac, renal, hematological, endocrine, or genetic metabolic diseases; (4) incomplete clinical information. The study was approved by the Ethics Committee of Sichuan Provincial Women’s and Children’s Hospital (No. 20231102–273). Informed consent was waived, as the research used retrospective, deidentified data extracted from an EHR database.

### 2.2 Data collection

Demographic data and prenatal examination information of the mothers were collected from the EHR, including the mothers’ age at childbirth, educational level, pregnancy complications (including anemia, gestational diabetes mellitus (GDM), hypertension during pregnancy (HDP), placenta or umbilical cord problems (PUC), and hypercoagulable state during pregnancy), and gestational weight gain (GWG). The infant’s gestational age, birth weight, delivery mode, sex, and feeding type were obtained from EHR.

We obtained physical measurement data and hematological test indicators from EHR. The physical measurement data of infants at 6 months of age, including weight and length, were collected by professionally trained nurses in strict accordance with the standard Anthropometric Measurements Method in Health Surveillance (WS/T 424–2013) (27). All measurements were conducted twice consecutively. If the discrepancy between the two measurements exceeded 20 g for weight or 2 mm for length, a third measurement was required. The average value of the two closest measurements was calculated (27).

Capillary blood samples were collected to test complete blood count (CBC) and serum ferritin (SF). CBC indicators include RBC, Hb, MCV, mean corpuscular hemoglobin (MCH), and mean corpuscular hemoglobin concentration (MCHC) detected by dual-acceleration flow cytometry. SF levels were determined using fluorescence immunochromatography. The reference intervals for MCV, MCH, MCHC, and SF were 80 ~ 100 fl, 27 ~ 34 pg., 320 ~ 360 g/L, and more than 20 μg/L, respectively (28).

### 2.3 Exposure

Infant sex was recorded at birth based on clinical examination and categorized as male or female, with female as the reference group in regression analyses.

### 2.4 Mediator

Weight gain in the first 6 months was calculated as the difference between the weight at 6 months and the birth weight (absolute growth increment). According to WHO Child Growth Standards (29) and the Chinese Medical Association Expert Consensus (30), absolute growth increment (change in weight over a specified period) is the standard metric for assessing cumulative growth in nutritional epidemiology. Using the median 0-6-month weight gain from our cohort (4.90 kg) as the cutoff, infants were classified into higher (≥4.90 kg) and lower (<4.90 kg) weight gain groups. In the primary mediation analysis, weight gain was analyzed as a continuous variable (kg) to maximize statistical power and avoid information loss. In the logistic regression analysis of factors associated with ID, the dichotomous classification was used to illustrate the risk contrast between higher and lower weight gain groups.

### 2.5 Outcome

Iron deficiency (ID) was defined as serum ferritin (SF) < 20 μg/L, based on a physiologically based cutoff proposed in the Lancet Haematology in 2021 (31). Iron deficiency anemia (IDA) was diagnosed when all the following criteria were met: Hb < 110 g/L, MCV < 80 fL, MCH < 27 pg., MCHC < 320 g/L, and SF < 20 μg/L (19). Due to the very low prevalence of IDA (5 of 355 infants), IDA was not analyzed as a separate outcome. All statistical analyses used ID (SF < 20 μg/L) as the primary outcome (*n* = 80), which encompassed both ID without anemia (*n* = 75) and IDA (*n* = 5).

### 2.6 Covariates

Maternal and infant characteristics included maternal education (College or above/High school or lower), anemia in pregnancy (Yes/No), HDP (Yes/No), PUC (Yes/No), delivery mode (vaginal delivery/cesarean section), GDM (Yes/No), hypercoagulable state (Yes/No), gestational age (weeks), GWG (kg), and feeding type. According to the World Health Organization definition of feeding (32), the classification criteria for feeding type were defined as follows: (1) exclusive breastfeeding: breastfeeding with no other food or drink, not even water; (2) mixed-feeding: mixture of breast milk and formula; (3) formula-feeding: fully formula-fed.

### 2.7 Statistical analysis

Statistical analysis was conducted using SPSS version 25.0 (IBM, Chicago, IL, USA). Continuous variables were assessed for normality using Shapiro–Wilk tests. Variables that were normally distributed are presented as means ± standard deviation (SD) and were compared between groups using independent-samples t-tests. In contrast, non-normally distributed variables were presented as medians (interquartile ranges [IQR]) and compared using Mann–Whitney U tests. Categorical variables are presented as frequencies and percentages [n (%)] and were compared using Pearson’s chi-square test or Fisher’s exact test as appropriate.

We examined the associations between maternal sociodemographic and obstetric characteristics and ID to screen for potential covariates using univariate logistic regression, with *p* < 0.15 used to select covariates for the subsequent adjusted model. Univariate logistic regression analyses were performed to estimate unadjusted odds ratios (ORs) with 95% confidence intervals (CIs) and screen potential risk factors for ID at 6 months. Separate multivariate models were then conducted to estimate adjusted ORs (aORs) for sex, feeding type, and weight gain, controlling for covariates. To determine the optimal weight metric, we evaluated multiple candidate variables (birth weight, 6-month weight, continuous and categorical weight gain) in univariate models. Continuous weight gain was selected for the final models based on its biological relevance to dynamic growth and maximal clinical interpretability. We further constructed a fully adjusted model that included male sex, feeding type, and continuous weight gain, along with all covariates to evaluate the mutually independent associations with ID risk.

To formally test whether weight gain mediated the association between male sex and ID risk, we performed mediation analysis using SPSS PROCESS macro (v4.1) with Model 4 (33). We first conducted separate regression analyses for each path: (1) the a path (sex → weight gain) used linear regression with results expressed as unstandardized *β* coefficients (kg difference); (2) the c path (total effect: sex → ID) used logistic regression unadjusted for weight gain; (3) the b path (weight gain → ID) used logistic regression adjusting for sex with weight gain modeled as a continuous variable (per kg increase); and (4) the c’ path (direct effect: sex → ID) used logistic regression adjusting for weight gain. All logistic regression results are presented as ORs with 95% CIs. The indirect effect (a × b) and its bias-corrected 95% CI were then estimated on the log-odds scale from 5,000 bootstrap samples, with significance determined by whether the CI excluded zero (34). The proportion mediated was calculated on the log-odds scale as (indirect effect / total effect) × 100. To examine whether the mediation effect differed by feeding type, we conducted stratified mediation analysis exclusively among breastfed infants (*n* = 189) using the same SPSS PROCESS macro (Model 4). For all other analyses, two-tailed *p* values <0.05 were considered statistically significant.

To examine potential effect modification, we conducted stratified analyses: (1) associations between sex and ID within each feeding type (exclusive breastfeeding, mixed feeding, formula feeding), and (2) associations between weight gain and ID by infant sex. Interaction terms (sex × feeding type and sex × weight gain) were tested to assess statistical heterogeneity. All stratified analyses used the same covariate adjustment as the main models.

## 3 Results

### 3.1 General characteristics of the study population

Among 2,282 infants who underwent both CBC and serum ferritin testing, 355 infants were eligible for final analysis after applying exclusion criteria (Figure 1). Participant characteristics are shown in Table 1. Of the 355 infants, 5 met criteria for IDA and 75 (21.1%) had ID without anemia. Combined ID (SF < 20 μg/L) was present in 80 infants (22.5%). Due to the very low IDA prevalence, subsequent analyses used the combined ID outcome (*n* = 80).

**Table 1:** Characteristics of the infants.

Variables	Total (*n* = 355)	Non-ID (*n* = 275)	ID (*n* = 80)	Statistic	*p*
Mother characteristics					
Maternal age, years, M (Q₁, Q₃)	29.00 (27.00, 31.00)	29.00 (27.00, 32.00)	28.00 (27.00, 31.00)	Z = −0.78	0.436
Education, *n* (%)					
College or above	185 (52.11)	149 (54.18)	36 (45.00)	χ^2^ = 2.09	0.148
High school or lower	170 (47.89)	126 (45.82)	44 (55.00)		
Anemia in pregnancy, *n* (%)					
No	238 (67.04)	184 (66.91)	54 (67.50)	χ^2^ = 0.01	0.921
Yes	117 (32.96)	91 (33.09)	26 (32.50)		
GDM, *n* (%)					
No	285 (80.28)	227 (82.55)	58 (72.50)	χ^2^ = 3.95	**0.047**
Yes	70 (19.72)	48 (17.45)	22 (27.50)		
HDP, *n* (%)					
No	341 (96.06)	264 (96.00)	77 (96.25)	χ^2^ = 0.00	1.000
Yes	14 (3.94)	11 (4.00)	3 (3.75)		
PUC abnormalities, *n* (%)					
No	341 (96.06)	265 (96.36)	76 (95.00)	χ^2^ = 0.05	0.822
Yes	14 (3.94)	10 (3.64)	4 (5.00)		
Hypercoagulable state, *n* (%)					
No	316 (89.01)	250 (90.91)	66 (82.50)	χ^2^ = 4.48	**0.034**
Yes	39 (10.99)	25 (9.09)	14 (17.50)		
GWG, kg, M (Q₁, Q₃)	12.50 (10.00, 15.00)	13.00 (10.00, 15.00)	12.00 (10.00, 14.62)	Z = −1.76	0.078
Delivery mode, *n* (%)					
Vaginal delivery	253 (71.27)	195 (70.91)	58 (72.50)	χ^2^ = 0.08	0.782
Cesarean section	102 (28.73)	80 (29.09)	22 (27.50)		
Infants characteristics					
Gestational age, week, M (Q₁, Q₃)	39.00 (38.00, 40.00)	39.00 (38.00, 40.00)	39.00 (38.00, 40.00)	Z = −1.64	0.101
Birth weight, g, Mean ± SD	3291.38 ± 343.66	3305.44 ± 349.64	3243.04 ± 319.63	t = 1.43	0.153
Sex, *n* (%)					
Male	197 (55.49)	142 (51.64)	55 (68.75)	χ^2^ = 7.35	**0.007**
Female	158 (44.51)	133 (48.36)	25 (31.25)		
Feeding type, *n* (%)					
Breast-feeding	189 (53.24)	134 (48.73)	55 (68.75)	χ^2^ = 10.71	**0.005**
Mixed-feeding	91 (25.63)	75 (27.27)	16 (20.00)		
Fomula-feeding	75 (21.13)	66 (24.00)	9 (11.25)		
Height-6 m, M (Q₁, Q₃)	67.00 (65.50, 68.55)	67.00 (65.40, 68.40)	67.00 (66.00, 69.00)	Z = −1.38	0.168
Weight-6 m, kg, M (Q₁, Q₃)	8.20 (7.55, 8.80)	8.20 (7.50, 8.70)	8.47 (7.70, 9.05)	Z = −2.43	**0.015**
Ferritin-6 m, M (Q₁, Q₃)	27.30 (20.75, 39.70)	32.04 (23.85, 44.22)	14.70 (11.73, 18.02)	Z = −13.62	**<0.001**
Weight gain, kg, M (Q₁, Q₃)	4.90 (4.33, 5.46)	4.84 (4.29, 5.38)	5.19 (4.60, 5.77)	Z = −3.28	**0.001**
Weight gain group, *n* (%)					
Lower	176 (49.58)	145 (52.73)	31 (38.75)	χ^2^ = 4.84	**0.028**
Higher	179 (50.42)	130 (47.27)	49 (61.25)		

t, t-test; Z, Mann–Whitney test; χ^2^, Chi-square test; SD, standard deviation, M, Median, Q₁, 1st Quartile, Q₃, 3st Quartile. Bold values indicate statistical significance (*p* < 0.05).

ID, iron deficiency; GDM, gestational diabetes mellitus; HDP, hypertension during pregnancy; PUC, placenta or umbilical cord problems; GWG, gestational weight gain.

Compared with the non-ID group, the ID group had significantly higher median weight gain (5.19 kg [IQR: 4.60–5.77] vs. 4.84 kg [IQR: 4.29–5.38]; *p* = 0.001) and were more likely to be male (68.8% vs. 51.6%; *p* = 0.007). Feeding type distributions differed between groups (*p* = 0.005), with the ID group showing higher exclusive breastfeeding rates (68.8% vs. 48.7%). The prevalence of GDM was higher in the ID group (27.5% vs. 17.5%; *p* = 0.047), as was the prevalence of a hypercoagulable state (17.5% vs. 9.1%; *p* = 0.034). Other characteristics are presented in Table 1.

### 3.2 Sex-specific characteristics and iron deficiency risk

At 6 months, compared with females, male infants (*n* = 197) had significantly lower ferritin and hemoglobin (both *p* < 0.001), greater weight gain (5.12 kg [IQR: 4.67–5.68] vs. 4.45 kg [IQR: 4.10–5.14]; *p* < 0.001) and higher ID prevalence (27.9% vs. 15.8%; *p* = 0.007). Other hematologic indices are detailed in Table 2.

**Table 2:** Sex-specific characteristics and iron deficiency by feeding type.

Variables	Total (*n* = 355)	Males (*n* = 197)	Females (*n* = 158)	Statistic	*p*
Hematology profiles at 6 months					
Hemoglobin, g/l, Mean ± SD	118.43 ± 7.91	117.46 ± 8.23	119.63 ± 7.33	t = −2.60	**0.010**
MCV, fl, Mean ± SD	77.19 ± 3.90	76.12 ± 3.91	78.53 ± 3.46	t = −6.06	**<0.001**
RBC, 10^12/l, M (IQR)	4.58 (4.37, 4.84)	4.64 (4.43, 4.88)	4.53 (4.32, 4.72)	Z = −3.00	**0.003**
MCH, pg., M (IQR)	26.00 (24.90, 27.00)	25.50 (24.50, 26.40)	26.50 (25.42, 27.30)	Z = −6.03	**<0.001**
MCHC, g/l, M (IQR)	334.00 (328.00, 339.00)	332.00 (326.00, 338.00)	335.50 (330.00, 340.00)	Z = −3.55	**<0.001**
RDW, %, M (IQR)	13.50 (12.80, 14.20)	13.70 (13.20, 14.40)	13.10 (12.60, 13.80)	Z = −5.57	**<0.001**
MCV/RBC, M (IQR)	16.87 (15.72, 18.09)	16.46 (15.24, 17.52)	17.51 (16.23, 18.48)	Z = −4.87	**<0.001**
Ferritin, ng/ml, M (IQR)	27.30 (20.75, 39.70)	24.20 (19.60, 36.10)	31.25 (22.60, 44.33)	Z = −3.48	**<0.001**
Weight gain, kg, M (IQR)	4.90 (4.32, 5.47)	5.12 (4.67, 5.68)	4.45 (4.10, 5.14)	Z = −6.81	**<0.001**
Feeding type, *n* (%)					
Breast-fed	189 (53.2%)	100 (50.8%)	89 (56.3%)	χ^2^ = 1.42	0.492
Mixed-fed	91 (25.6%)	55 (27.9%)	36 (22.8%)		
Formula-fed	75 (21.1%)	42 (21.3%)	33 (20.9%)		
ID, *n* (%)	80 (22.5%)	55 (27.9%)	25 (15.8%)	χ^2^ = 7.35	**0.007**
ID by feeding type, *n* (%)					
Breast-fed	55 (29.1%)	36 (36.0%)	19 (21.3%)	χ^2^ = 4.90	**0.027**
Mixed-fed	16 (17.6%)	12 (21.8%)	4 (11.1%)	χ^2^ = 1.72	0.190
Formula-fed	9 (12.0%)	7 (16.7%)	2 (6.1%)	—	0.283^a^

t, t-test; Z, Mann–Whitney test; χ^2^, Chi-square test; a, Fisher’s exact test; SD, standard deviation; M, Median; IQR, interquartile. Bold values indicate statistical significance (*p* < 0.05). ID, iron deficiency; MCV, mean corpuscular volume; RBC, red blood cell; MCH, mean corpuscular hemoglobin; MCHC, mean corpuscular hemoglobin concentration; RDW, red blood cell distribution width.

Stratified by feeding type, exclusively breastfed males exhibited the highest ID incidence (36.0% vs. 21.3% in breastfed females, *p* = 0.027). The sex disparity persisted in mixed-fed and formula-fed groups but did not reach significance. Feeding type did not significantly modify the sex-ID association (*P* for interaction = 0.805) (Supplementary Table 1).

### 3.3 Factors associated with iron deficiency

Risk factors associated with ID among infants are presented in Table 3. Covariate screening identified variables with *p* < 0.15 in univariate analysis (Supplementary Table 2) for inclusion in subsequent adjusted models: education, GDM, hypercoagulable state, gestational age, and gestational weight gain. After covariates adjustment (Model 2), male sex (aOR = 2.02, 95% CI: 1.18 to 3.47, *p* = 0.011), exclusive breastfeeding (aOR = 2.90, 95% CI: 1.33 to 6.30, *p* = 0.007), 6-month weight (aOR = 1.44 per kg, 95% CI: 1.11 to 1.89, *p* = 0.007), continuous weight gain (aOR = 1.66 per kg, 95% CI: 1.23 to 2.24, *p* = 0.001), and categorical weight gain (higher vs. lower, aOR = 1.94, 95% CI 1.13 to 3.32, *p* = 0.016) were significantly associated with ID risk. In the mutually adjusted model (Model 3), exclusive breastfeeding was most strongly associated with ID risk (aOR = 3.37, 95% CI: 1.52 to 7.48, *p* = 0.003), followed by weight gain (aOR = 1.63 per kg, 95% CI: 1.17 to 2.27, *p* = 0.004). In contrast, the association for male sex was attenuated and became non-significant (aOR 1.57, 95% CI 0.88–2.83, *p* = 0.129).

**Table 3:** Factors associated with iron deficiency among infants at 6 months.

Variables	Row %	Model 1		Model 2		Model 3	
		OR (95%CI)	*p*	OR (95%CI)	*p*	OR(95%CI)	*p*
Sex							
Female	15.8	1.00 (Reference)		1.00 (Reference)		1.00(Reference)	
Male	27.9	2.06 (1.21 ~ 3.50)	**0.007**	2.02(1.18 ~ 3.47)	**0.011**	1.57(0.88–2.83)	0.129
Feeding type							
Fomula-fed	12.0	1.00 (Reference)		1.00 (Reference)		1.00(Reference)	
Breast-fed	29.1	3.01 (1.40 ~ 6.46)	**0.005**	2.90 (1.33 ~ 6.30)	**0.007**	3.37(1.52 ~ 7.48)	**0.003**
Mixed-fed	17.6	1.56 (0.65 ~ 3.78)	0.319	1.51 (0.62 ~ 3.68)	0.370	1.46(0.58 ~ 3.64)	0.419
Birth weight	NA	1.00 (1.00 ~ 1.00)	0.153	1.00 (1.00 ~ 1.00)	0.395	—	
Weight-6 m	NA	1.37 (1.06 ~ 1.77)	**0.017**	1.44 (1.11 ~ 1.89)	**0.007**	—	
Weight gain, kg	NA	1.58 (1.19 ~ 2.11)	**0.002**	1.66 (1.23 ~ 2.24)	**0.001**	1.63(1.17 ~ 2.27)	**0.004**
Weight gain group							
Lower	17.6	1.00 (Reference)		1.00 (Reference)		—	
Higher	27.4	1.76 (1.06 ~ 2.93)	**0.029**	1.94 (1.13 ~ 3.32)	**0.016**	—	

Model 1: Unadjusted model. Model 2: Adjusted for maternal education, gestational diabetes, hypercoagulable state, gestational age, and gestational weight gain. Model 3: Mutually adjusted model including male sex, feeding type, and weight gain, with covariates as in Model 2. OR, odds ratio; 95% CI, 95% confidence interval. Bold values indicate statistical significance (*p* < 0.05).

### 3.4 Mediating role of 6-month postnatal weight gain in the sex-ID association

The associations between sex, WG, and ID were shown in Table 4. All three models were adjusted for the same set of covariates selected from Supplementary Table 2, with feeding type additionally adjusted as a potential confounder. Model 1 (linear regression) showed that male sex was independently associated with higher weight gain (*β* = 0.61 kg, 95% CI: 0.44 to 0.78, *p* < 0.001), explaining 18% of the variance (R^2^ = 0.18, *F* = 10.07, *p* < 0.001). Model 2 (logistic regression) demonstrated that male sex increased the odds of ID (aOR = 2.13, 95% CI: 1.23 to 3.70, *p* = 0.007) with moderate discrimination (C-statistic = 0.70, 95% CI: 0.63 to 0.76). Model 3, which added weight gain to Model 2, showed that the association between male sex and ID was attenuated (aOR = 1.57, 95% CI: 0.88 to 2.83, *p* = 0.129), while weight gain remained independently associated with ID risk (aOR = 1.63 per kg, 95% CI: 1.17 to 2.27, *p* = 0.004). The model discrimination modestly improved (C-statistic = 0.72 vs. 0.70; 95% CI: 0.66 to 0.78).

**Table 4:** Path analysis of sex, weight gain, and iron deficiency risk.

Predictors	Model 1 Outcome: WG (kg)		Model 2 Outcome: ID		Model 3 Outcome: ID	
	β (95% CI)	*p*	OR (95% CI)	*p*	OR (95% CI)	*p*
Model Path	X → M (a)		X → Y (c)		X → Y (c’); M → Y(b)	
Sex (Male)	0.61(0.44–0.78)	**<0.001**	2.13(1.23–3.70)	**0.007**	1.57(0.88–2.83)	0.129
WG (kg)	—		—		1.63(1.17–2.27)	**0.004**
Model fit	R^2^ = 0.18, F = 10.07, *p* < 0.001		C-statistic = 0.70(0.63–0.76), χ^2^ = 30.54, *p* < 0.001		C-statistic = 0.72(0.66–0.78), χ^2^ = 38.99, *p* < 0.001	
Sample size	*n* = 355		*n* = 355		*n* = 355	

WG, weight gain; ID, iron deficiency. Model 1: linear regression, Model 2 and Model 3: logistic regression; Model 1 and Model 2: adjusted for all variables with *p* < 0.15 in the univariate analysis listed in Supplementary Table 1, including education, GDM, hypercoagulable state, gestational age, gestational weight gain, and feeding type was also adjusted. Model 3: WG was added to Model 2. Bold values indicate statistical significance (*p* < 0.05).

**Figure 2:**
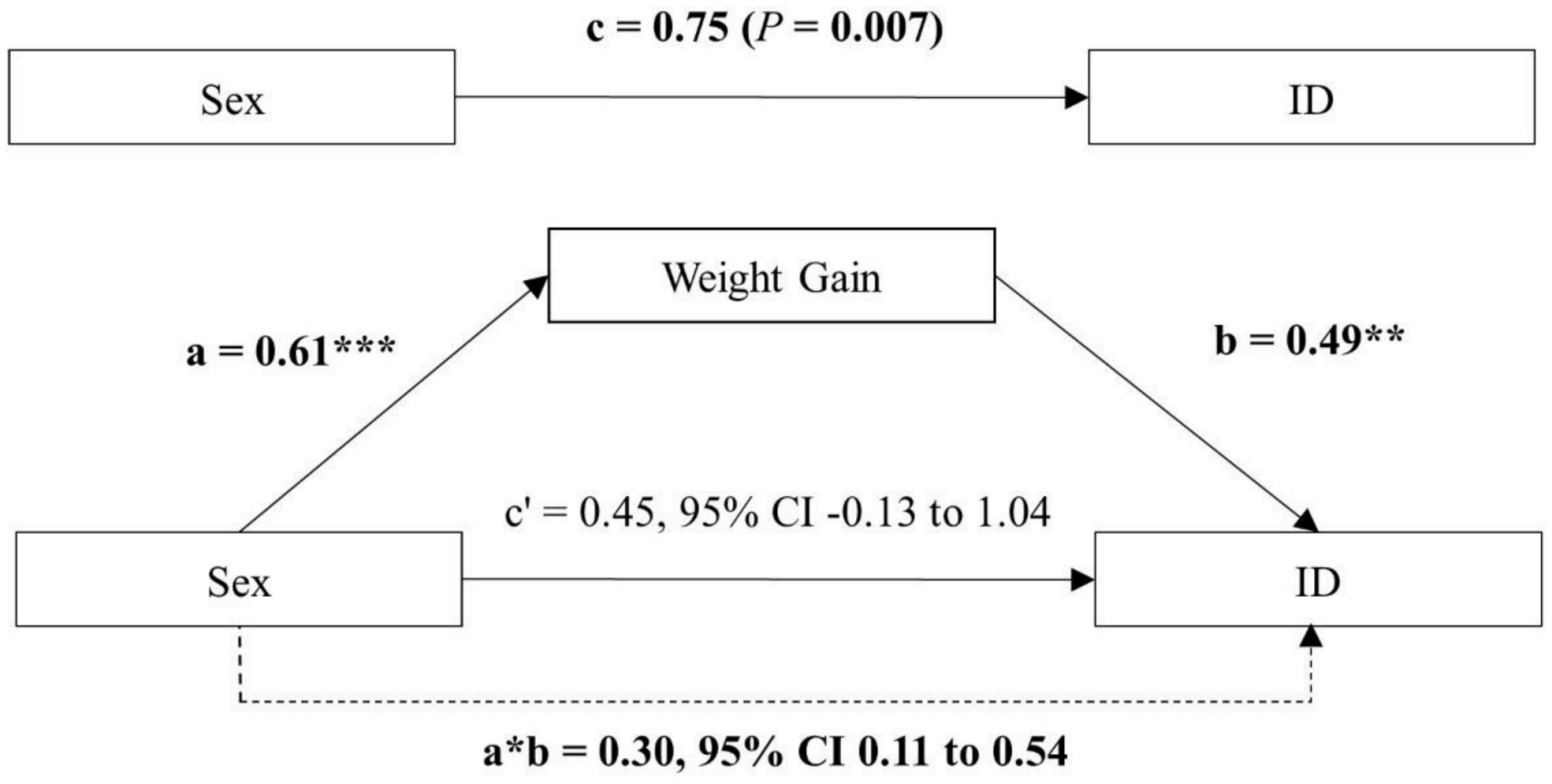
Mediation of weight gain at 6 months on the effect of sex on ID. The model is adjusted for education, GDM, hypercoagulable state, gestational age, gestational weight gain, and feeding type. *p < 0.05; ** *p* < 0.01; *** *p* < 0.001. C: total effect; c’: direct effect; a*b: indirect effect. Bold indicates significant values. ID: iron deficiency, WG: weight gain (kg).

We further examined the mediating role of weight gain, as illustrated in Figure 2. Weight gain significantly mediated the sex-ID association (indirect effect: 0.30, bootstrapped 95% CI: 0.11 to 0.54), accounting for 40% of the total effect. The direct effect of male sex on ID was attenuated and no longer statistically significant (0.45, 95%CI: −0.13 to 1.04). In the exclusively breastfed subgroup (Supplementary Figure 1), weight gain mediated 57% of the sex-ID association (indirect effect: 0.47, 95% CI: 0.18 to 0.90), with the direct effect similarly attenuated and non-significant (0.35, 95%CI: −0.39 to 1.10).

Furthermore, we examined whether the association between weight gain and ID differed by infant sex. As shown in Supplementary Table 3, while the interaction was not statistically significant (*P* for interaction = 0.608), weight gain was significantly associated with ID in male infants (aOR = 1.71, 95% CI: 1.15 to 2.54, *p* = 0.008) but not in female infants (aOR = 1.68, 95% CI: 0.89 to 3.16, *p* = 0.107), despite similar effect magnitudes.

## 4 Discussion

In this retrospective cohort of term, normal-birth-weight Chinese infants, we identified male sex, rapid weight gain during the first 6 months, and exclusive breastfeeding as independent risk factors for iron deficiency (ID) at the critical 6-month nutritional milestone. Our principal novel finding, derived from formal mediation analysis, reveals that accelerated weight gain explains 40% of the excess ID risk observed in male infants, with the direct effect of sex attenuating to non-significance after accounting for this growth-mediated pathway. These results provide timely evidence for ongoing debates regarding targeted iron supplementation strategies in early infancy, particularly highlighting the vulnerability of rapidly growing, exclusively breastfed male infants during the transitional period when endogenous iron stores become depleted.

ID during early life impairs neurodevelopment across cognitive, motor, and socio-emotional domains (15). In our cohort, the overall ID prevalence of 22.25% at 6 months, predominantly subclinical (21.1% without anemia, 1.4% with anemia), likely reflects our diagnostic threshold (serum ferritin <20 μg/L), which aligns with the Lancet Haematology (2021) physiologic standard subsequently adopted in Chinese national cohort studies (4, 35), though higher than the WHO <12 μg/L cutoff (28). Critically, global data for the 4–6-month window remain scarce, with prevalence ranging from 5.7% in Germany (4 months) to 21.4–36.4% in India (4–5 months) (36, 37); our 6-month estimate aligns closely with Indian 4-month data. The substantially higher ID prevalence (33.8%) reported in a 2024 Chinese national survey of infants aged 6–12 months (4), despite identical diagnostic criteria, highlights the 4–6-month period as a critical transition when endogenous stores deplete and dietary iron intake becomes inadequate.

Consistent with prior literature (15, 16, 38), our adjusted analyses confirmed that male sex, feeding type, and postnatal weight gain were individually associated with increased ID risk. Male infants exhibited approximately twice the ID risk of females (27.9% vs. 15.8%), corroborating previous observations of lower hemoglobin, mean corpuscular volume, and serum ferritin in males at 4, 6, and 9 months (11, 12). This sex disparity is further supported by national Chinese data showing higher ID prevalence in males (33.8%) than females (29.5%) among children aged 0.5–7 years (4). Regarding feeding practices, exclusively breastfed infants demonstrated markedly higher ID prevalence (29.1%) compared with mixed-fed (17.6%) and formula-fed (12.0%) groups (adjusted OR 2.9 versus formula-fed), consistent with Shao et al.’s finding of 4.73-fold higher ID odds in breastfed versus formula-fed infants at 9 months (39) and Yang et al.’s pooled estimate of 20% ID prevalence among exclusively breastfed infants (37). With 53% of our cohort exclusively breastfed, exceeding China’s reported rates of 29–34% and surpassing the World Health Assembly’s 2025 target, this high-risk population will continue expanding as policies drive toward the global goal of 60% exclusive breastfeeding by 2030 (40–42). The 4–6-month window represents a critical vulnerability for exclusively breastfed infants, as breast milk provides minimal iron (0.2–0.5 mg/L) insufficient to meet rapid growth demands after endogenous store depletion (16). Similarly, weight gain increment was positively associated with ID risk, aligning with Antunes et al.’s report of 1.6-fold increased risk per 1 kg gain during 0–9 months (13) and Lozoff et al.’s observation of increased ID incidence with rapid first-year growth (OR = 1.51) (15). Our study extends these findings to the 6-month window, demonstrating that 0–6-month weight gain, whether continuous (OR = 1.66) or categorical (OR = 1.94), significantly increased ID risk. Male infants exhibited accelerated growth compared with females (median 5.12 kg vs. 4.45 kg), consistent with 2022 Chinese national growth standards (25).

Notably, when all three factors were simultaneously included in multivariable models, feeding type and weight gain remained significantly associated with ID, whereas the association of male sex became non-significant. Further analysis revealed that sex differences in ID prevalence remained pronounced within the exclusively breastfed subgroup (36.0% in males vs. 21.3% in females). However, formal testing yielded a non-significant interaction between feeding type and sex on ID risk, suggesting insufficient statistical evidence that feeding type modifies the sex-ID association, though larger studies are warranted to confirm this.

Given the attenuation of the sex effect upon adjusting for weight gain, we conducted formal mediation analysis to test whether weight gain mediates the sex-ID relationship. Our results demonstrate that rapid weight gain explains 40% of the excess male ID risk, with the direct effect attenuating to non-significance (aOR 1.57, 95% CI 0.88–2.83, *p* = 0.129). This finding contrasts with earlier studies where male sex remained significantly associated with ID after adjusting for weight gain (13, 15). Of particular note, these studies employed more stringent diagnostic criteria (serum ferritin <12 μg/L) and observed higher ID prevalence (39.3–52.1%), suggesting that under more severe iron-deficient conditions, sex-specific biological pathways beyond weight growth may exert independent influences (13, 15). The biological mechanism likely involves hemodilution and iron kinetics: male infants’ greater weight gain (0.61 kg excess over 6 months) expands blood volume and tissue mass at rates exceeding hepatic iron mobilization and limited dietary iron availability (14). This interpretation aligns with Armitage et al.’s demonstration that rapid growth suppresses hepcidin, accelerating iron store depletion (14). Nevertheless, the remaining 60% of excess male risk and the attenuated but clinically relevant residual effect suggest additional mechanisms, including testosterone-mediated hepcidin suppression (43), differences in congenital iron stores (11) or sex-specific differences in iron absorption. In the exclusively breastfed subgroup, weight gain mediated 57% of the sex-ID association, substantially higher than the 40% observed overall, with the direct effect similarly becoming non-significant. This enhanced mediation likely reflects complete dependence on endogenous stores in exclusively breastfed infants, wherein male infants’ faster growth rapidly outpaces hepatic iron mobilization, leaving minimal residual sex-specific effects. These findings identify rapidly growing, exclusively breastfed male infants as a particularly vulnerable subgroup requiring early intervention.

Our results suggest that simple weight monitoring could enable early identification of high-risk infants for targeted intervention before 6 months, offering a precision approach superior to universal supplementation, which has proven relatively ineffective in reducing anemia burden (44). This ineffectiveness likely reflects mixing populations with differing absorptive capacities: infants with normal growth or adequate iron stores may have elevated hepcidin and impaired absorption, whereas rapidly growing infants with hepcidin suppression may be more responsive to supplementation (14). Furthermore, our findings emphasize the critical importance of timely introduction of iron-rich complementary foods before the 6-month milestone. However, real-world practices often deviate from recommendations: a Chinese national survey revealed only 32.2% of infants initiated complementary foods between 4 and 5 months (45), while US data indicated merely 38.3% started between 4 and 6 months (46). This gap exposes exclusively breastfed infants to prolonged ID risk during the vulnerable 4–6-month period. Clinical guidelines remain inconsistent: ESPGHAN does not recommend routine iron supplementation for healthy term exclusively breastfed infants (18), whereas updated Chinese expert consensus (2022–2023) recommends initiating supplementation at 4 months (1–2 mg/kg/day) for this population (10, 22). Given our findings and widespread delays in complementary feeding, further research is warranted to evaluate iron supplementation efficacy during the 4–6-month period.

Our study has several strengths. First, this study is the first to formally test weight gain as a mediator of the sex-ID relationship in term, normal-birth-weight Chinese infants at 6 months. Furthermore, comprehensive adjustment for feeding practices and prenatal confounders enhances robustness. However, several limitations should be acknowledged. First, as a single-center study from an urban, higher-socioeconomic-status setting (Chengdu), generalizability to rural populations may be limited. Second, the restriction to 6-month-old infants, capturing the critical window of endogenous iron store depletion, and the exclusion of infants receiving iron supplementation, may overestimate absolute ID risk compared to the general infant population, limiting generalizability to older infants and settings with routine supplementation. Third, despite rigorous exclusion of clinical infections, we lacked C-reactive protein or soluble transferrin receptor measurements to exclude subclinical inflammation, which may elevate SF and mask true deficiency. Fourth, while our sample size (*n* = 355) was adequate for primary mediation analyses, it lacked sufficient power to examine effect modification between feeding type and sex robustly. Consequently, we cannot definitively conclude whether the mediation effect differs across feeding types, necessitating large-scale future studies to test these interactions formally. Finally, the absence of infant iron status at birth (e.g., cord ferritin) represents a potential unmeasured confounder. Although we adjusted for gestational age, maternal education, and complications, neonatal iron stores independently predict later ID risk; their inclusion would have strengthened our analytical framework.

## 5 Conclusion

Our findings demonstrate that male sex is associated with higher ID prevalence at 6 months, with rapid weight gain accounting for 40% of this excess risk, rising to 57% among exclusively breastfed infants. Monitoring postnatal weight-gain trajectories, particularly among exclusively breastfed males, could guide early iron screening, timely introduction of iron-rich complementary foods, and pre-6-month targeted iron supplementation to mitigate ID and its associated neurodevelopmental risks. Future research should establish weight gain thresholds for clinical screening through large-scale cohort studies and conduct randomized controlled trials evaluating efficacy of iron supplementation specifically targeted to rapidly growing, exclusively breastfed male infants.

## Data Availability

The raw data supporting the conclusions of this article will be made available by the authors, without undue reservation.

## Glossary

ID: iron deficiency
WG: weight gain
CI: confidence interval
OR: odds ratio
WHO: World Health Organization
IDA: iron deficiency anemia
AAP: The American Academy of Pediatrics
ESPGHAN: The European Society for Pediatric Gastroenterology, Hepatology, and Nutrition
CBC: complete blood count
EHR: electronic health record
HAZ: height-for-age Z-score
WAZ: weight-for-age Z-score
WHZ: weight-for-height Z-score
Hb: hemoglobin
MCV: mean corpuscular volume
RBC: red blood cell
GDM: gestational diabetes mellitus
HDP: hypertension during pregnancy
PUC: placenta or umbilical cord problems
GWG: gestational weight gain
t: t-test
Z: Mann–Whitney test
χ^2^: Chi-square test
a: Fisher’s exact test
M: Median
Q₁: 1st Quartile,
Q₃: 3st Quartile
SF: serum ferritin
MCH: mean corpuscular hemoglobin
MCHC: mean corpuscular hemoglobin concentration
SD: standard deviation
IQR: interquartile ranges
ORs: odds ratios
CIs: confidence intervals
aORs: adjusted ORs
RDW: red blood cell distribution width.
