# Supplementary material for: Male sex and iron deficiency risk at 6 months: the mediating role of rapid weight gain
Source: Front Nutr. 2026 Apr 23;13:1829613. doi: 10.3389/fnut.2026.1829613 (PMC13149171; doi:10.3389/fnut.2026.1829613)
Supplement: Supplementary file 1 [file Table_1.docx]

Supplementary Material

**Supplementary Tables and Figures**

**Supplementary Table 1** Association between sex and iron deficiency stratified by feeding type

| **Variables** | ***n* (%)** | **Females** | **Males** | **OR (95%CI)** | ***P*** | ***P* for interaction** |
| --- | --- | --- | --- | --- | --- | --- |
| **All infants** | 355 (100.00) | 25/158 | 55/197 | 1.99 (1.16 ~ 3.42) | **0.012** |  |
| **Feeding type** | | | | | | |
| Breast-fed | 189 (53.24) | 19/89 | 36/100 | 1.98 (1.01 ~ 3.85) | **0.046** | 0.805 |
| Mixed-fed | 91 (25.63) | 4/36 | 12/55 | 1.89 (0.53 ~ 6.70) | 0.324 |  |
| Formula-fed | 75 (21.13) | 2/33 | 7/42 | 4.67 (0.76 ~ 28.56) | 0.095 |  |
| OR: Odds Ratio, CI: Confidence Interval. Bold indicates significant values (*p* < 0.05). Adjusted for maternal education, gestational diabetes, hypercoagulable state, gestational age, and gestational weight gain. | | | | | | |

**Supplementary Table 2 Covariates selection for iron deficiency**

| **Variables** | **Row %** | **uOR (95%CI)** | ***P*** |
| --- | --- | --- | --- |
| **Maternal age** | NA | 0.97 (0.91 ~ 1.04) | 0.368 |
| **Education** | | | |
| College or above | 19.5 | 1.00 (Reference) |  |
| High school or below | 25.9 | 1.45 (0.88 ~ 2.38) | 0.149 |
| **Anemia in Pregnancy** | | | |
| No | 22.7 | 1.00 (Reference) |  |
| Yes | 22.2 | 0.97 (0.57 ~ 1.66) | 0.921 |
| **GDM** | | | |
| No | 20.4 | 1.00 (Reference) |  |
| Yes | 31.4 | 1.79 (1.01 ~ 3.21) | **0.049** |
| **HDP** | | | |
| No | 22.6 | 1.00 (Reference) |  |
| Yes | 21.4 | 0.94 (0.25 ~ 3.44) | 0.919 |
| **PUC abnormalities** | | | |
| No | 22.3 | 1.00 (Reference) |  |
| Yes | 28.6 | 1.39 (0.43 ~ 4.57) | 0.583 |
| **Hypercoagulation** | | | |
| No | 20.9 | 1.00 (Reference) |  |
| Yes | 35.9 | 2.12 (1.04 ~ 4.31) | **0.037** |
| **Delivery** | | | |
| Vaginal delivery | 22.9 | 1.00 (Reference) |  |
| Cesarean section | 21.6 | 0.92 (0.53 ~ 1.61) | 0.782 |
| **Gestational age, week** | NA | 0.83 (0.65 ~ 1.07) | 0.149 |
| **GWG, kg** | NA | 0.94 (0.89 ~ 1.00) | 0.064 |

GDM, gestational diabetes mellitus; HDP, hypertensive diseases in pregnancy; PUC, placenta or umbilical cord problems; GWG gestational weight gain; uOR, unadjusted odds ratios; 95%CI, 95% confidence interval. Bold indicates significant values (*p* < 0.05). Variables with *P* < 0.15 in the univariate analyses were used as covariates in the subsequent adjusted model.

**Supplementary Table 3** Association between weight gain and iron deficiency stratified by infant sex

| **Variables** | ***n* (%)** | **OR (95%CI)** | ***P*** | ***P* for interaction** |
| --- | --- | --- | --- | --- |
| **All infants** | 355 (100.00) | 1.79 (1.32 ~ 2.45) | **<.001** |  |
| **Sex** | | | | |
| Female | 158 (44.51) | 1.68 (0.89 ~ 3.16) | 0.107 | 0.608 |
| Male | 197 (55.49) | 1.71 (1.15 ~ 2.54) | **0.008** |  |

OR: Odds Ratio, CI: Confidence Interval. Bold indicates significant values (*p* < 0.05). Adjusted for maternal education, gestational diabetes, hypercoagulable state, gestational age, gestational weight gain and feeding type.


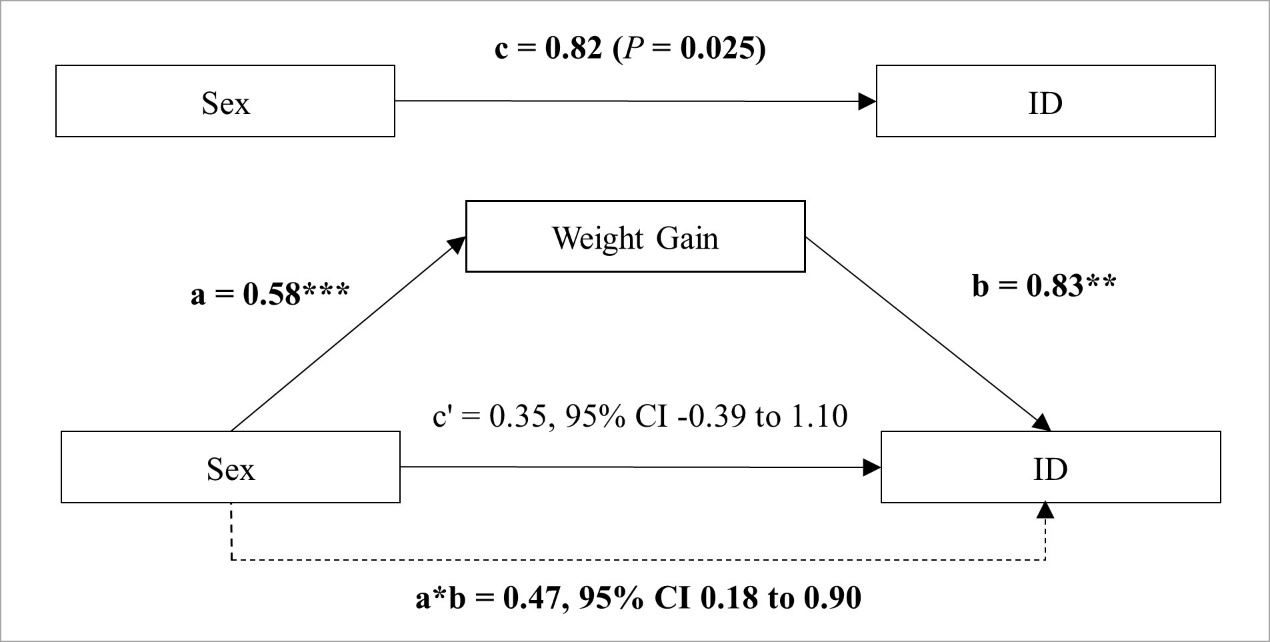


**Supplementary Figure 1** Mediation of weight gain at 6 months on the effect of sex on ID among exclusively breastfed infants. The model is adjusted for education, GDM, hypercoagulable state, gestational age, gestational weight gain. **P* < 0.05; ***P* < 0.01; ****P* < 0.001. c: total effect; c': direct effect; a*b: indirect effect. Bold indicates significant values. ID: Iron Deficiency, WG: Weight Gain (kg).
